# A Smartphone-Based Driver Safety Monitoring System Using Data Fusion

**DOI:** 10.3390/s121217536

**Published:** 2012-12-17

**Authors:** Boon-Giin Lee, Wan-Young Chung

**Affiliations:** Department of Electronic Engineering, Pukyong National University, Busan 608-737, Korea; E-Mail: leebgiin@pknu.ac.kr

**Keywords:** driver safety, eye features, bio-signal variation, Android-based smartphone, Fuzzy Bayesian, photoplethysmography, electrocardiography, temperature sensor, three-axis accelerometer, Bluetooth

## Abstract

This paper proposes a method for monitoring driver safety levels using a data fusion approach based on several discrete data types: eye features, bio-signal variation, in-vehicle temperature, and vehicle speed. The driver safety monitoring system was developed in practice in the form of an application for an Android-based smartphone device, where measuring safety-related data requires no extra monetary expenditure or equipment. Moreover, the system provides high resolution and flexibility. The safety monitoring process involves the fusion of attributes gathered from different sensors, including video, electrocardiography, photoplethysmography, temperature, and a three-axis accelerometer, that are assigned as input variables to an inference analysis framework. A Fuzzy Bayesian framework is designed to indicate the driver’s capability level and is updated continuously in real-time. The sensory data are transmitted via Bluetooth communication to the smartphone device. A fake incoming call warning service alerts the driver if his or her safety level is suspiciously compromised. Realistic testing of the system demonstrates the practical benefits of multiple features and their fusion in providing a more authentic and effective driver safety monitoring.

## 1. Introduction

Drowsiness is a multidimensional feature that researchers over the past decade have found difficult to define. Indeed, it is one of the leading contributing factors in traffic accidents worldwide. Solving the problem became critical when the design of earlier accident prevention systems was found ineffective for alerting the driver [1]. Therefore, a real-time fatigue detection system is essential in order to eliminate or reduce the risk of a driver having an accident. To develop drowsiness countermeasures, a greater understanding of driver fatigue in terms of its physiological properties is required [2,3]. The spectral analysis of the drowsiness state can be classified into a few discrete categories: the driver’s facial and body motion and physiological status (heartbeat, pulse rate); the vehicle’s operating condition; the in-vehicle environment; the driver’s driving aptitude or behavior (lane-keeping, speeding, anger, anxiety); and a combination of these.

Ji *et al.*[4] predicted driver fatigue levels using a probabilistic model based on a visual cues model that included eyelid movement, gaze movement, and head motion. Kumar *et al.*[5] presented a methodology for driver drowsiness detection using tracking of the pupils’ motion. The driver is determined to be fatigued only if the eyes are closed for several consecutive frames within a specific time period; otherwise, the driver is blinking his or her eyes, and a diagnosis of fatigue would be false. A vision system is proposed by Yao *et al.*[6] to measure the driver’s vigilance level by integrating a number of facial parameters, including those of the eyes, mouth, and gaze. Lee *et al.*[7] proposed a system that uses two fixed cameras to capture images of the driver and the road, respectively. The images are then mapped to global coordinates to monitor the driver’s sight line. These systems require extra cameras to be installed to capture driver facial images. Our proposed system utilizes the smartphone front camera without any vehicle modification, especially power wiring or external cameras, to perform eye detection task.

On the other hand, Kecklund *et al.*[8] stated that recorded EEG signals demonstrate a significant intra-individual correlation between subjective sleepiness and alpha burst activity. Lin *et al.*[9] proposed a real-time brain-computer interface (BCI) system to monitor human physiological and cognitive states by analyzing EEG signals. They demonstrated that the amplitude of an EEG peak value, which is estimated by a drowsiness detection system, may predict a driving error. Wang *et al.*[10] proposed a latent variable to represent the attributes of individual drivers to recognize their emotional state using four sensors, specifically, respiration, blood pressure, skin conductance and temperature sensors. Yang *et al.*[11] used a first order Hidden Markov Model (HMM) to compute the dynamics of a Bayesian Network (BN) for compiling information to infer the level of driver fatigue by analyzing multiple physiological characteristics, such as ECG and EEG signals. In order to measure EEG signals, sensors must be placed at the forehead or other parts around the brain. In [29], the ECG electrodes are placed at the driver seat and measurements are carried out when the driver’s back side is in contact with the electrodes, but ECG signals can’t be measured if the driver seat cloth is too thick as the contact distance between body and electrodes is increased. Meanwhile, Yang *et al.*[30] integrated the sensors in a wearable shirt to measure ECG signals. Most drivers are not willing to wear such shirts during driving as it may feel uncomfortable and most importantly the shirt is difficult to clean and wash. Our proposed system avoids such limitations by integrating the circuit on the steering wheel. ECG signals are measured from fabric electrode when hands are placed on the fabric.

Zhao *et al.*[12] studied the reliability of steering behavior analysis for detecting driver fatigue by applying a support vector machine (SVM) model using a multi-wavelet packet energy spectrum. Pauwelussen *et al.*[13] developed a traffic-simulation model in which a vehicle is equipped with an adaptive cruise-control (ACC) and a lane-departure warning (LDW) system to monitor driver behaviors in real traffic environments. Wang *et al.*[14] introduced a dangerous-driving warning system that uses statistical modeling to mine the safe/dangerous driving patterns from time-series data with very limited labeling information. Although the labeling information is targeted only for accidents, the learned model is able to predict non-crash dangers, such as a near miss or other dangerous driving maneuvers. Moreover, Liang *et al.*[15] developed an in-vehicle system to detect driver distraction that applies SVMs, trained on all the data collected in simulator experiments. Three factors were investigated: how distraction can be defined; which data should be input to the model; and how the input data should be summarized. The above systems predict the driver vigilance index based on driving behavior and vehicle movement instead of driver actual condition in real-time. The alertness prediction is more accurate by analyzing on the driver facial features and biomedical signals.

In addition, environmental factors, such as temperature, light, vibration, *etc.*, are considered capable of influencing a driver’s drowsiness. Landstrom *et al.*[16] evaluated the efficiency of temperature variation measurements in a vehicle as an indication of the driver’s drowsiness. The application’s results indicated that the chances of drowsy driving occurring can be greatly reduced by maintaining a cooler temperature in the vehicle. Li *et al.*[17] investigated the effect of an innovative cooling device that is intended to make subjects more alert and less sleepy, and found that induced temperature shifts may reduce the body’s capability to enter the sleep state. It is clear that most people cannot fall into a drowsy state when the atmosphere is frigid, and sleep symptoms are more likely in a warm atmosphere. Besides temperature, vehicle speed is also crucial for avoiding accidents. Moreno *et al.*[18] combined steering wheel movement data, acceleration and braking data, and speed of the vehicle for detecting that the driving pattern of a driver indicates drowsiness. The first results of the research were promising; however, Moreno concluded that more elaborate driving tests must be performed to evaluate and adjust the system for optimal performance. Dillies *et al.*[19] used a real-time fuzzy pattern recognition process implemented in a neural network whose input is signals from the steering wheel, a sensor that measures the speed of the car, and the accelerator. Briefly, the parameters of these external factors such as speed and temperature can contribute to predict the driver’s vigilance index and is well implemented in our proposed system.

Even though existing drowsiness monitoring systems perform well, they have limitations in terms of their approach. For instance, methodology that uses facial features requires a long moving-averaged window to track slow changes in a driver’s vigilance. In the case of bio-signal processing, existing techniques require that sensors be attached to the human body to obtain vital signs. This could distract the driver and cause discomfort. Finally, in order to study driver-vehicle interaction, the vehicle’s structure has to be modified, which is impractical and unwise in a real assessment.

The focus and objective of this study was to develop a reliable, well-controlled and non-intrusive [31] drowsiness monitoring system that comprises the following aspects: (1) fusion of attributes or data that are obtained from sensory data to derive an accurate drowsiness prediction; (2) implementation of a multi-functional monitoring system in an Android-based smartphone device; (3) integration of a Fuzzy Bayesian network in a smartphone device to predict the driver’s aptitude and alertness state over time; (4) a low-cost solution for capturing the driver’s image using the front-facing video sensor of a smartphone device; and (5) a reliable fake incoming call type alert system to warn and wake the driver without generating adverse effects on the driver.

## 2. System Design

**Figure 1.**
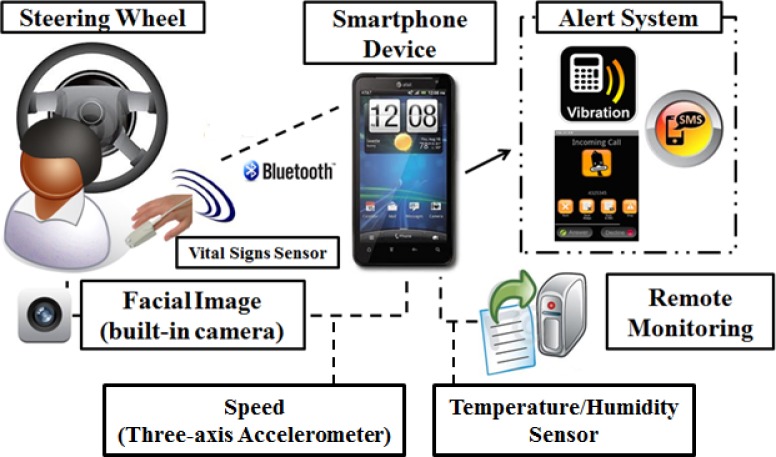
System design overview consists of several modules where smartphone device receives data from ECG, PPG and humidity sensors that placed on the steering wheel. Driver facial image is captured via smartphone front camera and vehicle speed is calculated with built-in three-axis accelerometer. Alert system is triggered if prediction vigilance index reached over 75 (0 is lowest and 100 is highest).

This section describes the backbone of the system, which comprises several modules. Basically, these are: a facial features motion module; a bio-signals spectral analysis module; an inference paradigm framework module; a smartphone device module; and a fake call alert module. Figure 1 illustrates an overview of the system design for the proposed system. Essentially, the smartphone device receives sensory data via biomedical sensors that are attached on the steering wheel, and also the driver’s facial images via the smartphone’s built-in front-facing camera. In addition, the system optimizes a built-in sensor to gather the required sensor readings. The three-axis accelerometer reading from a built-in accelerometer sensor can be used to measure the speed of the vehicle. Temperature data can be obtained via a humidity sensor, which is placed on the steering wheel so that the temperature can be measured as close as possible to the driver to obtain an optimal reading. An extraction process is performed to extract meaningful features from the received data; these features then serve as input models to an inference network to analyze the driver’s vigilance level. The network predicts the driver’s alertness state through a series of computations, and displays the computed results on the smartphone’s screen. An alert system is triggered if the statistical results indicate that the driver’s alertness is predicted to be low.

The extraction of sensory data is further explained in the following subsection.

### 2.1. Eyes Feature Extraction

Feature extraction can be divided into two main processes: template-matching and color-based extraction. The template matching technique finds small parts of an image by matching it to a template image, while color information can be used to estimate the facial area that matches a certain skin color. The color-based approach is more suitable for application in a smartphone device because this approach does not require as large a database as template-matching does. In fact, the detection accuracy depends only on the type of skin color to be matched.

**Figure 2.**
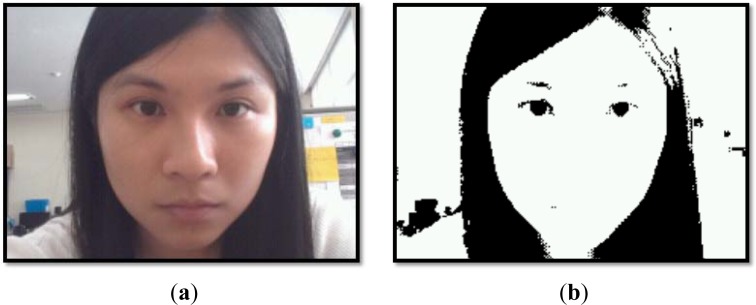

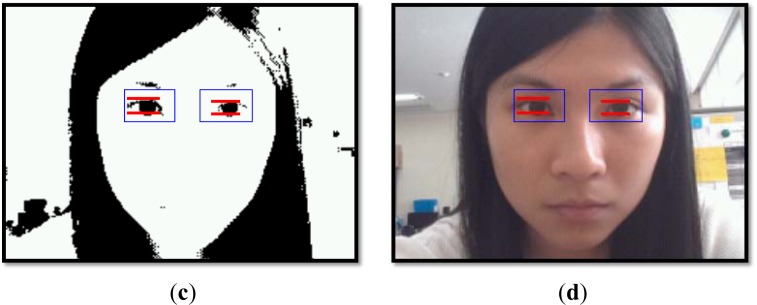
Eyes detection using the HRV color model approach. (**a**) Original face image captured by smartphone front video-sensor. (**b**) Converted binary threshold image. (**c**) The eyes location is indicated using the facial region; the eyes are located in the upper part and are vertically synchronized with each other, with an error tolerance of ±10 pixels. (**d**) Location of eyes in original image.

The HSV [20], also known as HSI, color space is a good indicator for locating a facial area given an input image. *H* stands for hue and refers to the degree to which a stimulus can be described as similar to or different from other stimuli, which are red, green, blue, and yellow. *S* stands for saturation, which is the combination of the hue and lightness values, and V stands for value (*I* stands for intensity). In the Android platform, the image data buffer is encoded in YUV format. First, the YUV image is converted into the HSV color space. Once a converted HSV image has been obtained, its binary threshold is calculated based on its hue index, and the image is converted further into a black and white image, known as a binary image. The morphological close operation is performed to smooth the border areas, and any holes outside the large boundary areas are then removed. Based on the border that surrounds the black region, a facial region is extracted. Figure 2 shows an example of face and eyes detection based on the HSV color model. Once the binary threshold image is obtained, the regions of both eyes can be identified inside the white regions (with the boundary surrounded by the black region). Normally, both eyes are located on a vertical line. However, if two identical scenarios are found, such as eyebrows and eyes, as shown in Figure 2(b), the boundary that is located in the lower part of the facial region and is larger has a higher confidence level of being eyes. The proposed smartphone application is presented in landscape mode to avoid any distraction to the driver if smartphone is placed at the back of steering wheel. However, the smartphone can be placed at the left or right side near to steering wheel as long as the smartphone front camera is able to capture the driver face image.

### 2.2. Bio-signal Feature Extraction

**Figure 3.**
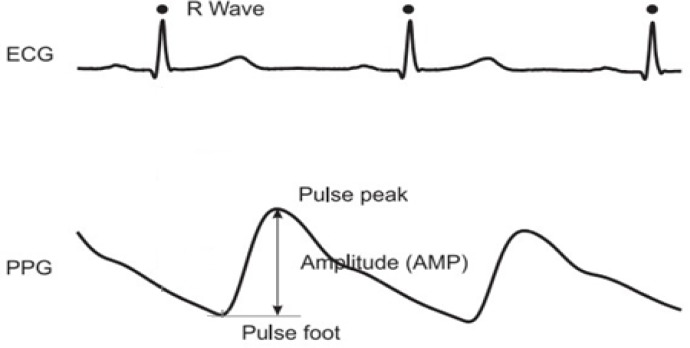
Waveform of PPG and ECG signals in which ECG is the manifestation of heart contractile activity while PPG is signals that measure the changes in skin blood volume and perfusion. These signals are further extracted into useful parameters as inputs to inference network for vigilance prediction.

The proposed system also indicates the driver’s fatigue level, using electrocardiography (ECG) and photoplethysmography (PPG) signals collected from an in-door driving simulation as its training set. The PPG signal system [21] is a non-invasive optical technique that measures changes in skin blood volume and perfusion. It contains components that are synchronous with respiratory and cardiac rhythms. This technique measures changes in skin blood using a light probe that is placed on the surface of the skin. Meanwhile, the ECG signal is the manifestation of the contractile activity of the heart, which is a valuable indicator of the individual’s overall activity level [22]. It is the recording of the electrical activity on the body surface generated by the heart. Figure 3 illustrates the waveform of PPG and ECG signals, respectively.

### 2.3. Features Measurement Method

The extracted features can be used further to predict the driver’s drowsiness level by observing the feature variation over time. The percentage of eyelid closure (PR) [23] over the pupil over time can reflect slow eyelid closure (“droops”) rather than blinks to predict driver drowsiness. Here, the duration of the eyelid closure refers to the percentage of eyelid closure over a specific time and has P70, P80, EM three measurement methods. P80 refers to 80% of the largest pupil size and is considered the best indicator of a driver’s drowsiness. A fatigued driver should have longer eyelid closure duration, since he or she blinks distinctly more slowly than an alert driver.

Another indicator is heart rate variability (HR). It is as a known physiological phenomenon that the time interval between heart beats varies. This indicator can be obtained by measuring the R wave to R wave (RR) interval of ECG signals. Another fatigue analysis indicator is blood pressure (BP), sometimes referred to as arterial blood pressure, which is the pressure exerted by circulating blood upon the walls of blood vessels, and is one of the principal vital signs. Hayashi *et al.*[24] studied ambulatory blood pressure for 24 hours in white-collar workers. They showed that subjects who worked a large amount of overtime (mean 84 hours/month) had higher blood pressure, slept fewer hours, and were more fatigued before and after work than subjects who worked a smaller amount of overtime (mean 26 hours/month), whose hypertension was only mild. It seems that the blood pressure can be used to infer the vigilance index of a driver. BP is calculated from PPG signals as defined in Equation (1):

(1) BP=(a*PP)−b

Here, ***PP*** is the interval between PPG peak and valley in a single cycle measured in a time unit. Meanwhile, ***a*** and ***b*** are the constant values calculated using a calibration method. Different people may exhibit different values, but for flexibility purposes, we used the mean values of ***a*** and ***b***, respectively, based on the training dataset. The calibration technique is outside the scope of this research; further details are given in [25].

As opposed to the values above, the temperature value is fed directly from the humidity sensor, no additional conversion or computation being required. In order to measure the speed (SP) of the vehicle, a smartphone built-in three-axis accelerometer is utilized. The calculation of speed in a km/h measurement unit is defined in Equation (2):

(2) $$\begin{array}{c} \bar{\mathrm{SP}}=\sqrt{x\_{\mathrm{axis}}^{2}+y\_{\mathrm{axis}}^{2}+z\_{\mathrm{axis}}^{2}}\\ \mathrm{SP}=\left(\bar{\mathrm{SP}}-C\right)*3.6 \end{array}$$

where $\bar{\mathrm{SP}}$ is the non-calibrated speed value, while ***x_axis***, ***y_axis*** and ***z_axis*** is the reading of the accelerometer for the x, y and z axis, respectively. ***C*** is the standard gravity value of 1 g or can be defined precisely as 9.80665 m/s^2^, or about 35.30394 (km/h)/s.

## 3. Fuzzy Bayesian Network

The proposed Fuzzy Bayesian network (FBN) is a Bayesian network, the variables of which have fuzzy states. A Bayesian network [26] is a directed acyclic graph (DAG) that represents a joint probability distribution among a set of variables. Nodes are the variables and the connected links among the nodes are the conditional dependencies among variables. Each node is associated with a probability function that takes a particular set of values as inputs for the node’s parent variables and gives the probability of the variable represented by node. Those dependencies are characterized by a conditional probability table (CPT). In order to set up a Bayesian network, the first step is to specify the nodes of the discrete network. The second step is to specify what features are used to represent the discrete variables while the final step is to configure the initial states of variables for calculating the Bayesian network.

The concept of fuzzy sets is used to aid the estimation of entropies (uncertainty). It provides a basis for a qualitative approach to the analysis of complex system that employs linguistic rather than numerical variables to describe system behavior and performance. The fuzzy rule expression is close to an expert natural language. A fuzzy system manages the uncertain knowledge and infers higher level of behavior from the observed data. Each fuzzy variable defines the membership degree [heart rate (**HR**), blood pressure (**BP**), temperature (**TP**), speed (**SP**) and PERCLOS (**PR**)] to output state, fatigue: as the value of fatigue closer to 1, the higher prediction of being drowsy, and vice versa.

In a Bayesian network, although all the variables defined are discrete, most are genuinely continuous. According to the axioms of the probability theory, the discrete states of an originally continuous variable must be mutually exclusive and collectively exhaustive. Therefore, since the cut between two neighboring discrete states is often hard to define, it is difficult to quantize the mapping from the continuous value domain to the discrete state frame logically. FBN combines the fuzzy knowledge of a variable state with uncertainty. Finally, the integration of fuzzy logic and a Bayesian network can adapt the advantages of both representations. Further details of fuzzy logic and Bayesian network are presented in [26]. The indicator ***y*** can be defined as in Equation (3), where ***x*** is the total ***n*** members in fuzzy states, while ***k*** denotes the probability distribution of the respective members:

(3) $$y=\left\{x_{a}^{1},x_{b}^{2},x_{c}^{3},\ldots ,x_{k}^{n}\right\}$$

Belief propagation in FBN is quite similar to that in a Bayesian network. In real-time analysis, the fuzzy values of input variables can be obtained through the membership functions. The five indicators, **HR**, **BP**, **TP**, **SP** and **PR** are the parent nodes of the output variable, fatigue in FBN. The integration of parent components can be measured using the Cartesian product in which the membership functions for each variable can be calculated using the product *t-norm*[27]. The **fatigue** can be calculated based on a conditional probability table (**CPT**) and fuzzy values, as shown in Equation (4), assuming that the fuzzy values of the four indicators have only true and false values:

(4) $$\mathrm{fatigue}=\left(\sum_{1}^{p=L}\bar{t\_{\mathrm{norm}}_{p}}\times \bar{{\mathrm{cpt}}_{p}},\sum_{1}^{p=L}t\_{\mathrm{norm}}_{p}\times {\mathrm{cpt}}_{p}\right)$$

Here, ***L*** is the total number of possible combinations available, and $\bar{t\_\mathrm{norm}}$ and $\bar{\mathrm{cpt}}$ refer to the product t-norm and the CPT that denotes the false value, respectively. The fatigue is classified with three output states which are denoted as “safe”, “warning” and “dangerous”. Each fuzzy state has an FBN probability which define how high the possibility of the state being is. For instance, if state “safe” shows probability of 0.2, it denotes that this state has probability of 0.2 is being the current driver vigilance state. Therefore, the current driver vigilance state can be defined as the state that possesses the highest probability value. In summary, the calculation complexity of the FBN output probability depends on the number of fuzzy members in each membership function, as well as the number of elements (nodes) in the Bayesian network.

## 4. Experimental Setup

**Figure 4.**
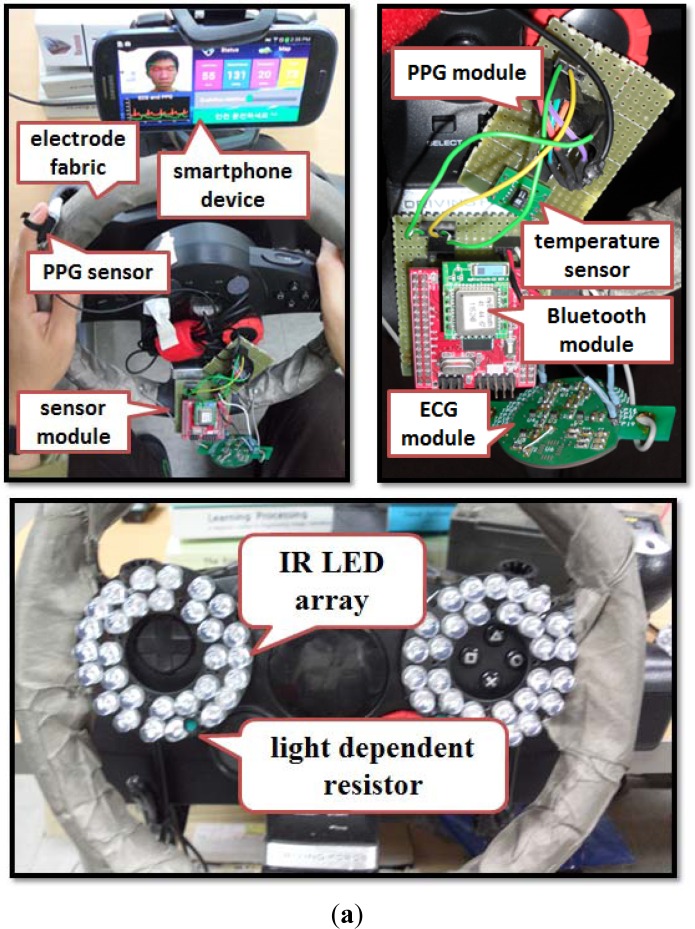

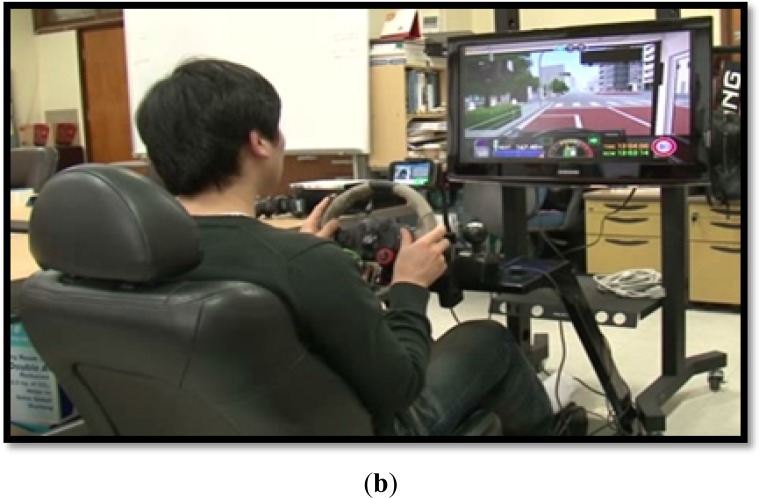
(**a**) ECG, PPG and temperature sensor modules placed on a steering wheel where the sensor data are transmitted to the smartphone device via Bluetooth communication. PPG signals are measured by placing a finger on the sensor while ECG signals are measured by placing both hands on the electrode fabric (integrated on steering wheel). Infra-red lights array with a light dependent resistor (to detect light intensity) can be placed on the steering wheel or other regions to enable clear capture of facial images during night driving. (**b**) Experiment scenario for dataset collection and application testing with 3D driving simulation, a steering wheel for driving control and acceleration and brake pedals.

Figure 4(a) depicts the installation of ECG, PPG and temperature sensor modules on a steering wheel, which are connected to an Atmega128 microprocessor with a Bluetooth module attached on it; the ECG, PPG and temperature data packets are transmitted to the smartphone device through a Bluetooth connection. The smartphone device used in our proposed system is the Samsung Galaxy S III (http://www.samsung.com/global/galaxys3; accessed on 17 September 2012). The experiments were simulation-based, where the smartphone device was placed behind the steering wheel, and the 3D driving simulation was displayed in front of the driving seat.

## 5. Results and Discussion

The fatigue evaluation performed by the proposed FBN inference network is displayed in Table 1. The FBN inference results revealed a high rate of true awake state prediction for subject E, and an accurate and true drowsy state prediction for subjects D, F, and G, representing an approximately 99% accuracy rate. In contrast, the test subsets showed the lowest rate of true awake state prediction (94%) for subjects A and J, and the lowest rate of true drowsy state prediction (94%) for subjects A and B. The true awake or drowsy state predictions of the FBN, which represent an accuracy rate greater than 95%, are considered satisfactory, whereas the other predictions, such as those for subjects A, B, and J are considered unsatisfactory. Even though the average rate of true predictions for either the awake or drowsy state was relatively high, the rate of false estimations was moderately high as well, being greater than 5% for subjects A, B, and J. Finally, the average rates of true awake state predictions and true drowsy state predictions were 96% and 97%, respectively. Despite these results, the results of the fatigue analysis using the FBN inference model are promising, since a higher complexity yielding a higher accuracy of the inference network might slow down the overall processing of the smartphone device, which is not appropriate for long-term use.

In the process of fatigue prediction, a safety monitoring system must be able to notify a driver within a very short time period when a dangerous situation arises. Wakasugi [28] observed that drivers proceed to change lanes when the time before a collision would otherwise occur is at least six seconds. It was recognized that a lower threshold might be required if a driver returns to the lane very quickly following a warning. The overall system response time is approximately two seconds. However, an accident might already have occurred if the system issued the warning to the driver within two seconds. In this developed application, the change in the driver’s condition from non-partial sleep to partial sleep can be recognized in approximately 0.2 s. It is shown that for a fully awake subject, the FBN probabilities are maintained at an interval value between 0.30 and 0.54. If the probability interval value of the FBN increases to 0.60, it is considered that the driver is starting to get drowsy. The FBN probability at an interval value between 0.60 and 0.75 is defined as the threshold for signifying the driver is in the partial-sleep condition, and the driver will be advised to rest. Once the probability has reached a value greater than 0.75, a warning service is triggered to alert the driver.

Table 2 summarizes the lane departure systems available in the market for different vehicle manufacturers. Almost all the lane warning systems employed similar methods by either mounting video cameras or infra-red sensors to detect lane markings on the road. Very little research has been published on the evaluation of lane change assistance systems in this field of expertise. This may be due to sensitivities surrounding advanced commercially produced systems. There may also be a reluctance to sponsor such research due to the difficulty in assessing in experiments whether the system has prevented a collision. At the present time, very few field tests or simulation studies on lane change assistance systems exist. Further research is required on the effects of lane change assistance and the potential for unintended consequences.

Meanwhile, Table 3 illustrates the overview of existing drowsiness systems developed by researchers. Some researchers are focusing on identifying the regions in which fatigue or drowsiness may occur but are less concentrated on identification of performances in the long term. Others, such as [4] demonstrated the system performance based on each defined parameter, but did not combine them to develop a trustful fatigue detections system. Therefore, the proposed system has greater advantages than do the existing systems in various aspects. First, the proposed system is not easily affected by external factors. For instance, in the above systems, the infrared sensors or cameras are not able to detect lane markings reliably and correctly under heavy rain conditions. Moreover, these systems also performed badly when the road was covered with thick snow or mud. In addition, so the sensors or cameras can be mounted, vehicle structure modifications, especially in terms of electrical wiring, is essential. These extra tools and accessories are usually expensive and most drivers have no intention of spending extra money on such modifications. Furthermore, the smartphone applications are much more easily updated than hardware (sensors) of a vehicle due to their different life cycle and lifespan. Extra efforts are required to change or replace sensors in the vehicle if a sensor integration inside the vehicle approach is being adopted. The commercial products are focused basically on the driving behavior instead of the physical state of the driver. In addition, the lane marking evaluation system can be operated only at speeds of between 60 and 250 km/h and only after the system had detected a lane marking. In summary, the proposed developed system is not constrained by the limitations mentioned above and is able to perform comprehensive fatigue analysis.

**Figure 5.**
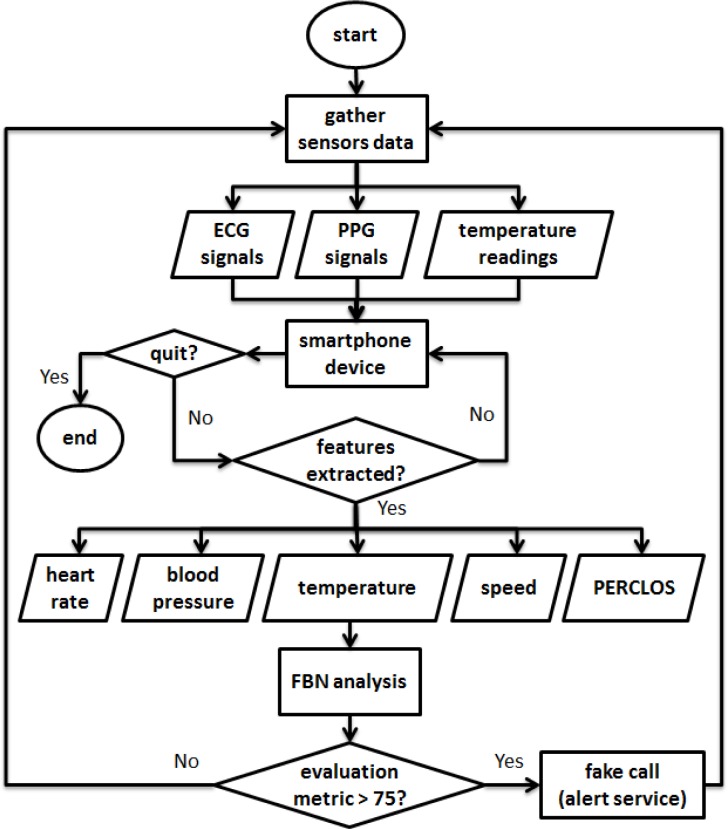
Application flowchart where the application first converts the sensors reading into useful parameters which serves as inputs to Fuzzy Bayesian network and the fake call service is triggered if predicted vigilance metric is over 75 (0 is lowest and 100 is highest).

Figure 5 illustrates the flow chart for the application developed in a smartphone device. Firstly, the application received the ECG, PPG and temperature readings from the respective sensors. Then, features or parameters are derived including heart rate, blood pressure, temperature, speed and PERCLOS which are extracted from ECG, PPG, humidity readings, three-axis accelerometer values and driver facial image respectively. If the FBN has probability of over 0.75 (75%), fake call service is generated to warn the driver with ringtone and vibration enabled.

Table 4 denotes the time computation for each processing in the developed application. The application spends longest times in the vigilance prediction process using FBN analysis network which is approximately 0.1 s. Each variable extraction takes around 0.01 s depending on the data buffer size. If the buffer contains less than fifteen data points, there is the likelihood that more than two peaks or valleys can be detected. The screen update is near to 0.05 s, while sensor reading and encoding conversion takes less than 0.001 s. The raw sensor readings are converted from a byte array to hexadecimal encoding and finally into decimal values.

**Figure 6.**
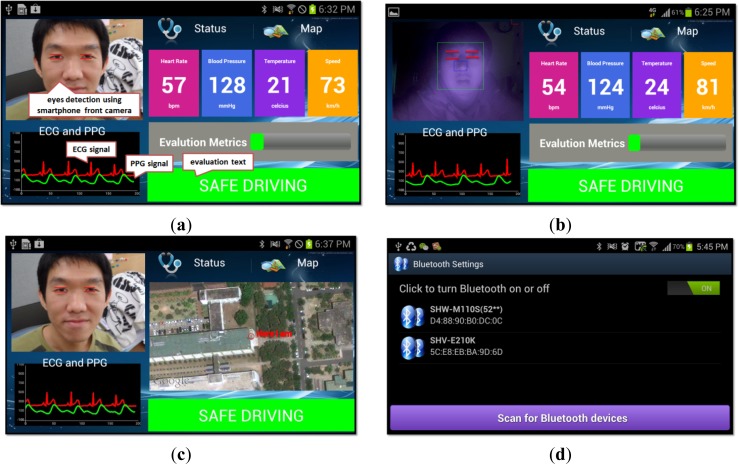

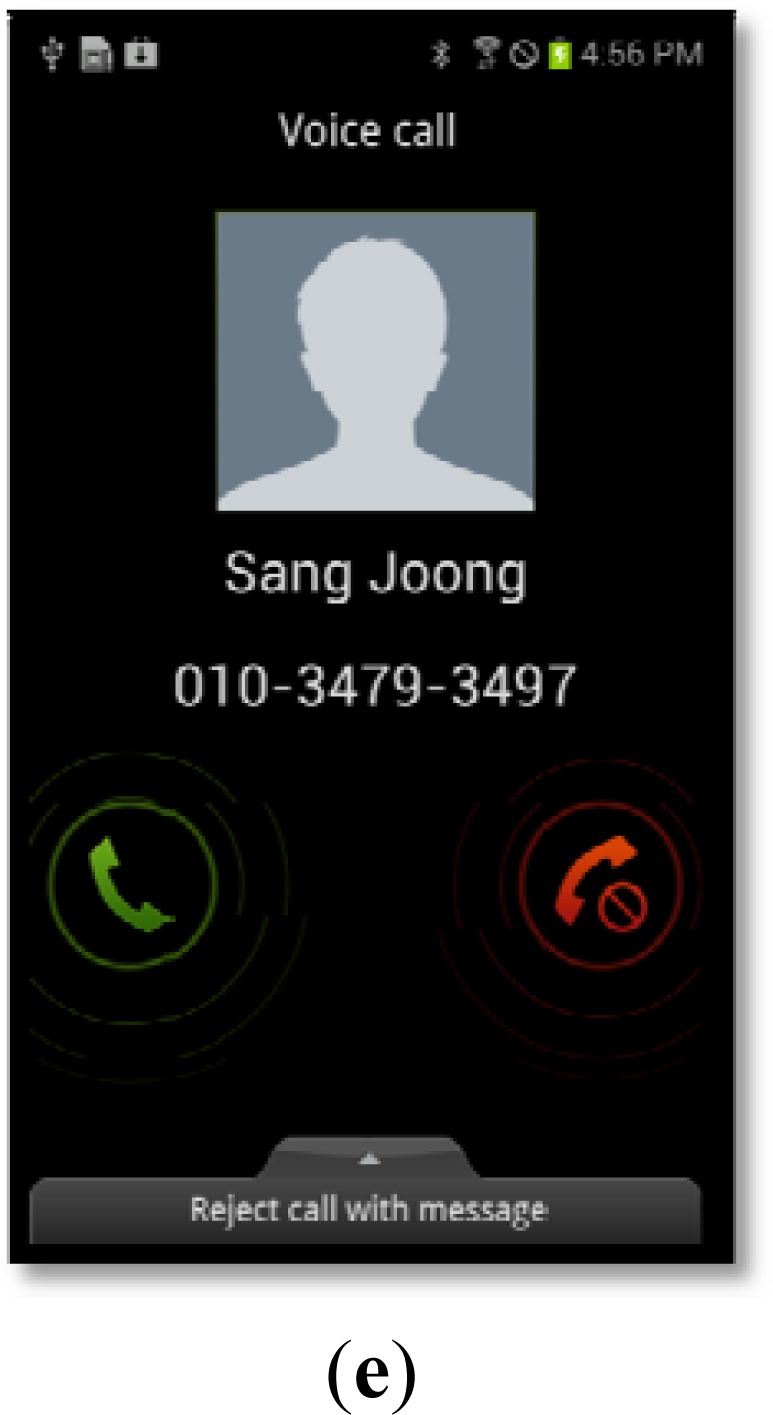
Screenshots captured in a real Android-based smartphone device. (**a**) Main screen for safety monitoring application with real-time eyes detection, plotting of ECG and PPG signals, parameters update include heart rate, blood pressure, temperature, speed as well as vigilance evaluation. (**b**) Demonstrates the face and eyes detection at night using IR lights. (**c**) A map navigation service is included to aid the driver so that an additional GPS monitor for driving guidance is not required. (**d**) Bluetooth settings menu to connect to the sensor modules to receive sensor data. (**e**) Fake incoming call alert service to warn the driver if the evaluation metric has reached over 75%.

Figure 6 demonstrates a few screenshots of the prototype system, which was developed on the Android platform. Figure 6(a) shows the main screen of the system that performs fatigue analysis in real-time, and Figure 6(b) shows the capability of our proposed system to perform face and eyes detection during night time with the integration of IR lights. Figure 6(e) shows the fake call generator’s main configuration screen, which allows the caller’s name and phone number, and messages for the text messaging service to be modified.

## 6. Conclusions

A real-time non-invasive driver fatigue detection system for application in a smartphone device has been designed and developed. The focus was on feature fusion in which several distinct features or parameters are integrated, including heart rate variability (HR), blood pressure (BP), temperature (TP), speed (SP), and percentage of eyelid closure (PR). A Fuzzy Bayesian network (FBN) is implemented to predict and analyze the driver’s vigilance index. Once the evaluation metric reaches 75% (in FBN probability expressed as 0.75), a fake call service is initiated along with a loud ringtone and maximum vibration strength to alert the driver of his/her current dangerous driving state. Moreover, the application provides several configurable options for the driver. For example: the vigilance evaluation can be enabled or disabled; the user can choose which features are used in the evaluation; and the caller name, phone number, vibration (enables or disable), ringing time duration, and ringtone type can be changed.

## Figures and Tables

**Table 1.** FBN inference network performances for ten subjects.

**Tester**	Total of Test Samples		Total of True Prediction		Total of False Prediction		True (%)		False (%)	
	**AW**	**DW**	**AW**	**DW**	**AW**	**DW**	**AW**	**DW**	**AW**	**DW**
**A**	126	142	118	133	8	9	94%	94%	6%	6%
**B**	182	155	176	146	6	9	97%	94%	3%	6%
**C**	146	137	141	132	5	5	97%	96%	3%	4%
**D**	167	166	161	164	6	2	96%	99%	4%	1%
**E**	181	171	179	165	2	6	99%	96%	1%	4%
**F**	157	166	150	164	7	2	96%	99%	4%	1%
**G**	186	175	178	174	8	1	96%	99%	4%	1%
**H**	153	149	148	145	5	4	97%	97%	3%	3%
**I**	163	181	155	176	8	5	95%	97%	5%	3%
**J**	144	173	135	165	9	8	94%	96%	6%	4%
**Total**	**1605**	**1615**	**1541**	**1564**	**64**	**51**	**96%**	**97%**	**4%**	**3%**

**AW–awake samples, DW–drowsy samples.**

**Table 2.** Lane departure systems available on market.

**OEM**	**System**	**Technology (Video Camera)**	**Trigger Speed**	**Driver Alert**
**Lexus**	LDW	Mounted behind windscreen monitors vehicle position in relation to lane markings		Audio-visual Warning
**After Market**	SafeTRAC	Mounted behind windscreen monitors vehicle position in relation to lane markings		Visual Lane Position Display
**Mercedes**	SPA	Mounted behind windscreen monitors coach position in relation to lane markings	80 km/h (50 mile/h)	Vibrating Driver’s Seat
**Nissan**	LDW	Mounted behind windscreen monitors vehicle position in relation to lane markings	72 km/h (45 mile/h)	Dashboard Display & Audible
**Audi/VW**	Lane Assist	Mounted behind windscreen monitors vehicle position in relation to lane markings	65 km/h (41 mile/h)	Steering Wheel Vibration
**BMW**	LDW	Mounted behind windscreen monitors vehicle position in relation to lane markings	70 km/h (44 mile/h)	Dashboard Display or Steering Wheel Vibration
**GM**	LDW	Mounted behind windscreen monitors vehicle position in relation to lane markings	56 km/h (35 mile/h)	Audible & Visual
**Citroen**	LDWS	Six pairs of infra-red sensors at front of car	80 km/h (50 mile/h)	Vibrating Driver’s Seat
**Volvo**	LDW	Mounted behind windscreen monitors vehicle position in relation to lane markings	64 km/h (40 mile/h)	Audible

**Table 3.** Overview of existing drowsiness detection system.

**System**	Sensors/Parameters	Algorithm	Accuracy
**[1]**	EEG, ECG	Mean power frequency	-
**PODS [3]**	Respiration Rate, Heart Rate, Heart Rate Variability	Power Spectrum	-
**[4]**	Cameras/Eyelid movement, gaze movement, gaze movement, head movement and facial expression	Kalman filtering tracking	Yawn–82% PERCLOS–86% AECS–95%
**[5]**	IR Camera	Thresholding, Mean	-
**[6]**	Camera/facial features of eyes, mouth and head	Fuzzy reasoning	Only focused on detection rate for facial tracking and face tracking rate
**BCI [9]**	EEG	Principal Component Analysis (PCA)	Training–92.6% Testing–74.6%
**[11]**	ECG, EEG	dynamic Bayesian network, first-order Hidden Markov Model	Drowsy (best)–91% Active (best)–91%
**IVIS [15]**	Eye movement, driving performance data	Support Vector Machines (SVMs)	Distraction detection (average)–81.1%
**Proposed System**	Smartphone (display and front camera), ECG, PPG	Fuzzy Bayesian network	True Awake–96% True Drowsy–97%

**Table 4.** Application processing time.

**State**	**Time (s)**
Sensors Reading and Encoding Conversion	0.001
Eyes detection and PERCLOS calculation	0.050
ECG R-R wave detection and heart rate derivation	0.010
PPG peak-to-valley detection and blood pressure derivation	0.010
Temperatures reading and speed derivation	0.020
FBN analysis	0.100
Update display screen	0.005
Total	0.196
